# Reverse Engineering a Signaling Network Using Alternative Inputs

**DOI:** 10.1371/journal.pone.0007622

**Published:** 2009-10-29

**Authors:** Hiromasa Tanaka, Tau-Mu Yi

**Affiliations:** 1 Department of Developmental and Cell Biology, University of California Irvine, Irvine, California, United States of America; 2 Center for Complex Biological Systems, University of California Irvine, Irvine, California, United States of America; Center for Genomic Regulation, Spain

## Abstract

One of the goals of systems biology is to reverse engineer in a comprehensive fashion the arrow diagrams of signal transduction systems. An important tool for ordering pathway components is genetic epistasis analysis, and here we present a strategy termed Alternative Inputs (AIs) to perform systematic epistasis analysis. An alternative input is defined as any genetic manipulation that can activate the signaling pathway instead of the natural input. We introduced the concept of an “AIs-Deletions matrix” that summarizes the outputs of all combinations of alternative inputs and deletions. We developed the theory and algorithms to construct a pairwise relationship graph from the AIs-Deletions matrix capturing both functional ordering (upstream, downstream) and logical relationships (AND, OR), and then interpreting these relationships into a standard arrow diagram. As a proof-of-principle, we applied this methodology to a subset of genes involved in yeast mating signaling. This experimental pilot study highlights the robustness of the approach and important technical challenges. In summary, this research formalizes and extends classical epistasis analysis from linear pathways to more complex networks, facilitating computational analysis and reconstruction of signaling arrow diagrams.

## Introduction

Arrow diagrams are the lingua franca of molecular biologists. Although such diagrams may possess different meanings [1], [2], the semantics for signal transduction arrow diagrams tend to be better defined. A pointed arrow (→) indicates the activation of a target by an activator species, and a blunt arrow (−−|) represents the inhibition of the target by an inhibitor. The diagram traces the pathway from the input(s) to the output(s). Typically these arrow diagrams are assembled in a piecemeal fashion from the discoveries of different labs. For example, the ordering of the yeast pheromone pathway has been determined through the work of several labs over several years [3]. A challenge for systems biology is developing more systematic methods for constructing these diagrams.

There are several large-scale resources in budding yeast including the genome sequence [4], single deletion libraries [5], double deletion (synthetic lethal) libraries [6]–[8], gene expression arrays [9], overexpression libraries [10], whole genome two-hybrid studies [11], [12], affinity purification libraries [13], [14], the localization of proteins based on GFP-tagged proteins [15], ChIP-chip data for transcription factor binding information [16], and gene annotations (*Saccharomyces* Genome Database; http://www.yeastgenome.org/). These resources offer a vast amount of information about the functions and interactions in the whole genome-wide system. A recent exciting approach is epistatic miniarray profiling (E-MAP) [17] which assesses in a quantitative fashion the genetic interaction between two loss-of-function mutations. However, one drawback of all of the above methods is the absence of a direct interpretation into a standard arrow diagram. For example, the positive or negative genetic interaction between two genes does not specify a direct functional relationship without additional information [18].

Theoretical and computational methods to reverse engineer signaling networks have been developed using genome-wide proteomic, expression, and deletion data, and these techniques employ Boolean methods, mutual information, Bayesian inference, regulation matrix methods based on differential equations, and machine learning approaches (reviewed in [19], [20]). Generally speaking these approaches rely on sophisticated inference methods to combine different sources of information to reconstruct the network. The work of Van Driessche et al. [21] and E-MAP [22], [23] are elegant genetic epistasis techniques, but these studies used loss-of-function deletion mutant combinations, and so they too relied on sophisticated indirect approaches to infer the arrows. The classic epistasis analysis used here with gain-of-function/loss-of-function combinations directly determines whether or not an arrow exists between two genes with logical relationships (i.e. AND or OR) between two genes. We believe that the loss-of-function/loss-of-function approaches and our gain-of-function/loss-of-function approach can complement one another.

**Figure 1:**
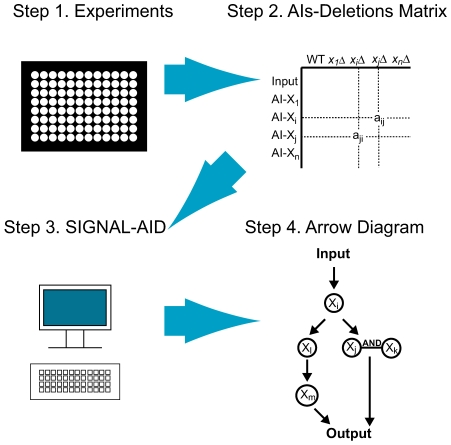
Schematic flow chart for reverse engineering a signaling network using alternative inputs. In Step 1, experiments are performed to measure the outputs of all combinations of gain-of-function alternative inputs and loss-of-function deletions, as well as the natural input and the wild-type background. In Step 2, we create the AIs-Deletions matrix using the experimental data in Step 1. In Step 3, we analyze the AIs-Deletions matrix using the software package SIGNAL-AID, which constructs an arrow diagram for the signaling network (Step 4).

Here, we developed the infrastructure and assessed the feasibility of performing systematic epistasis analysis on a large-scale (e.g. genome-wide). We term this approach “Alternative Inputs” and define an “Alternative Input (AI)” to be any genetic manipulation that can activate the signaling pathway instead of the natural input [24]. Overexpression of an activator would be a typical alternative input. Central is the concept of an “AIs-Deletions matrix”, which captures all possible combinations of gain-of-function alternative inputs and loss-of-function deletions summarizing the results of a systematic epistasis experiment. This matrix is converted into a pairwise relationship graph that provides not only functional ordering (upstream, downstream) but also logical relationships of molecules (AND, OR) that expand the analysis beyond linear pathways to branched networks. We have then devised algorithms to use this relationship information to reconstruct a signaling pathway in standard arrow diagram form (Figure 1). We named this software SIGNAL-AID (Software for Identifying Genetic Networks with Arrows and Logics by Alternative Inputs and Deletions). We applied the alternative inputs methodology to the yeast mating signaling system as a proof-of-principle. This pilot study revealed technical challenges as well as robustness in the approach. We propose that systematic epistasis analysis and the data collected in an AIs-Deletions matrix can complement current functional genomics approaches.

## Results

### Alternative Inputs (AIs) and AIs-Deletions Matrix

We start with the notion of a signal transduction network with a natural input (e.g. ligand) and a measured output (e.g. transcriptional reporter). This system can be represented by a signaling arrow diagram in which a pointed arrow from gene/protein X_i_ to X_j_ denotes that X_i_ activates X_j_. An “Alternative Input (AI)” is defined as any genetic manipulation that can activate the signaling pathway and output instead of the natural input. For activators, the alternative input would be the overexpression of the wild-type or constituitively-active form of the gene. For repressors, the alternative input would be a gene deletion (Text S1, Figure S3 and S6).

**Figure 2:**
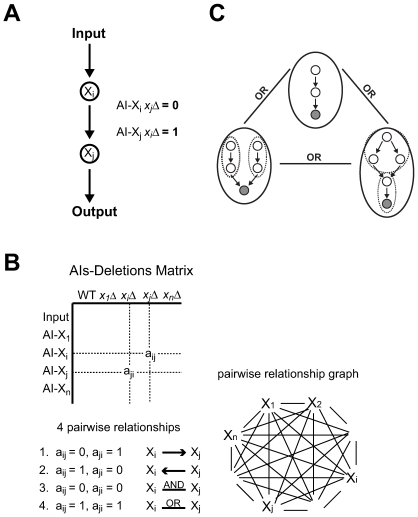
Reconstructing a signaling arrow diagram using the AIs-Deletions matrix. (A) Epistasis analysis using alternative inputs. The double mutant combinations of *AI-X_i_ x_j_Δ* and *AI-X_j_ x_i_Δ* indicate that X_i_ is upstream of X_j_. (B) The concept of an AIs-Deletions matrix. An AIs-Deletions matrix describes outputs by the original input and alternative inputs (rows) in a wild-type strain and their corresponding deletion strains (columns) in a combinatorial manner. The entry *a_ij_* contains the output for cells with the genotype *AI-X_i_ x_j_Δ*. There are four possible pairwise relationships between X_i_ and X_j_ as specified by the elements *a_ij_* and *a_ji_*: 1) X_i_ is upstream of X_j_, 2) X_i_ is downstream of X_j_, 3) X_i_ AND X_j_, and 4) X_i_ OR X_j_. These relationships form the edges of a fully-connected pairwise relationship graph. (C) Recursive decomposition of OR-Included relationship graphs. After identifying AND nodes, the software SIGNAL-AID decomposes the graph by identifying the largest subgraphs in which all nodes share a common downstream node (C-node, shaded). After this step, we are left with a reduced graph of C-node subgraphs (within solid ovals) that are fully connected by OR-edges (3-OR in this example). Each C-node subgraph can be recursively decomposed to smaller subgraphs (dashed ovals) and ultimately individual nodes in a similar fashion by identifying common downstream nodes.

Ordering in a pathway can be determined by classic genetic epistasis analysis [3]. For example, if X_i_ activates X_j_ produces the output, then *AI-X_i_ x_j_Δ* (strain containing the alternative input X_i_ and the deletion of X_j_) would produce no output, whereas *AI-X_j_ x_i_Δ* would produce an output response (Figure 2A). Thus, the phenotype of the double mutant combination determines the upstream/downstream ordering. One can imagine performing epistasis analysis in a more systematic fashion by making all possible combinations of AIs and deletions. We formalized this idea with the concept of an “AIs-Deletions matrix” (Figure 2B). Here we refer to a “deletion” as a genetic perturbation that blocks signaling through the system. The convention is that the rows contain the natural input (first row) followed by the different AIs, and the columns contain the wild-type background (first column) followed by the different deletions. Thus, matrix element *a_ij_* = Output (*AI-X_i_ x_j_Δ*). By setting a threshold, we can convert this real-valued matrix into a Boolean AIs-Deletions matrix **B**, consisting of 1's (output on) and 0's (output off). Finally, we refer to the submatrix 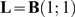 as the local (Boolean) AIs-Deletions matrix i.e. the submatrix without the first row (natural input) and column (wild-type).

### Pairwise Relationship Graph

A key theoretical concept is that of the pairwise relationship graph (Figure 2B). Each pair of elements (*a_ij_*, *a_ji_*) in the local Boolean AIs-Deletions matrix describes the relationship between molecule X_i_ and molecule X_j_. The elements can take the values (*a_ij_*, *a_ji_*) = (1, 0), (0, 1), (1, 1), or (0, 0), and each value pair describes one of four types of genetic interactions between the signaling molecules X_i_ and X_j_: (a) (0, 1) = X_i_ is upstream of X_j_; (b) (1, 0) = X_i_ is downstream of X_j_; (c) (0, 0) = X_i_ AND X_j_; and (d) (1, 1) = X_i_ OR X_j_ (Figure 2B). One interpretation of the AND relationship is that X_i_ and X_j_ form a functional complex; an interpretation of the OR relationship is that X_i_ and X_j_ are in parallel pathways. These logical relationships extend the epistasis analysis beyond linear pathways to branched networks.

### OR-Excluded AIs-Deletions Matrix

The next step described below is transforming the pairwise relationship graph into a signaling arrow diagram. First we will consider pairwise relationship graphs without any OR edges, i.e. OR-excluded graphs. We will also assume that there are no cycles; one interpretation of a cycle is a positive feedback loop which should result in an AND relationship among the nodes (Supplementary Information). The resulting pairwise relationship graph consists of directed edges and AND edges.

The initial step is to remove the AND edges by collapsing two nodes connected by an AND edge into a joint AND node e.g. X_i__AND_X_j_. After this preprocessing, only directed edges remain in a linear chain. One can determine the ordering of this chain by iteratively identifying the most downstream node and then connecting that node with the previous most downstream node.

### Or-Included Graphs and the Complete k-OR Graph

From a biological standpoint, an OR-edge in the pairwise relationship graph indicates the presence of parallel signaling pathways. Such a parallel pathway in the signaling arrow diagram arises from a branch node in which a protein activates more than one target protein. OR-edges greatly increase the complexity of the transformation of a pairwise relationship graph into an arrow diagram. Below, we describe one algorithmic approach to the problem.

As before, we first identify the AND edges and create joint AND nodes. The remaining edges in the pairwise relationship graph are the upstream/downstream arrows and the OR edges. Then, we decompose the graph by identifying the largest subgraphs in which all nodes share a common downstream node; we represent these subgraphs by their common downstream node or C-node. After this step, we are left with a reduced graph of C-nodes that are fully connected by OR-edges (k-OR graph if there are *k* C-nodes). Each C-node subgraph can be recursively decomposed using this procedure until we are at the level of individual nodes (Figure 2C). The processes described above were implemented in a software package termed “SIGNAL-AID (Software for Identifying Genetic Networks with Arrows and Logics by Alternative Inputs and Deletions)”.

### Enumeration of Arrow Diagram Structures Arising from a k-OR Group

**Figure 3:**
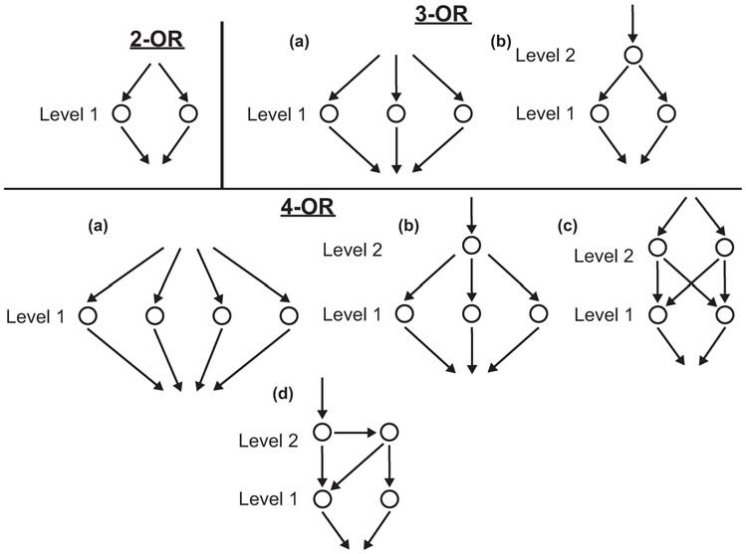
Enumerating minimal arrow diagram structures for 2-OR, 3-OR, and 4-OR graphs. The topologies for the diagrams are described in terms of the number of nodes at each Level; a Level L node is *L* edges from the Output. In the 2-OR case, there is a single topology and one diagram structure. In the 3-OR case, there are two topologies and two diagrams. In the 4-OR case there are three distinct topologies (4 Level 1 nodes; 3 Level 1 nodes and 1 Level 2 node; 2 Level 1 nodes and 2 Level 2 nodes) and 4 diagrams; there are two different diagram structures for the 2 (Level 2), 2 (Level 1) topology.

A group of *k* nodes possessing a mutual OR relationship can give rise to many legitimate arrow diagrams. However, one can simplify the feasible space by considering only the diagrams with a minimum number of directed edges. Here we describe a procedure for enumerating these minimal graphs (Figure 3).

We classify the diagrams in terms of levels, which are defined by their distance from the common downstream node e.g. Output node. Different topologies possess different numbers of nodes at the different levels. Level 1 indicates nodes that directly connect to the Output; Level 2 describes nodes that connect to Level 1 nodes but not directly to the Output. A Level L node is a minimum of *L* edges from the Output.

We start with the 2-OR case and then add nodes. In the 2-OR case we have a single topology consisting of 2 Level 1 nodes. To construct the 3-OR case, we can add a Level 1 node to create 3 Level 1 nodes, or add a Level 2 node to the 2-OR case leading to 1 Level 2 node and 2 Level 1 nodes. Continuing in this fashion, we can list the 3 4-OR topologies.

The next step is to connect to the nodes in each topology. The Level 1 nodes connect to the common downstream node (or Output). Each node in Level 2 possesses 2 directed edges. These connections can be made to either a node on the next lower level or to a node on the same level. All possibilities are enumerated. Thus, there are 4 3-OR minimal diagrams, because one topology (2 Level 2 nodes, 2 Level 1 nodes) gives rise to two distinct minimal arrow diagram structures.

One can generalize this approach to list the minimal arrow diagrams for an arbitrary k-OR case. The complexity increases significantly for 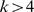, but the analysis is beyond the scope of this report. In addition, one can identify *Min+x* representations by taking the minimal diagrams and adding *x* extra edges.

### Using Data to Select among Possible k-OR Arrow Diagrams

**Figure 4:**
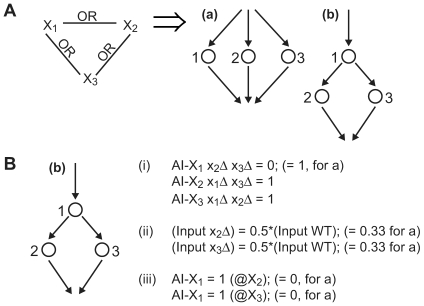
Using additional information to distinguish between minimal 3-OR arrow diagrams. (A) Shows the two possible minimal arrow diagrams for the 3-OR case (reproduced from Figures 3). The three nodes are labeled 1, 2, and 3. (B) Additional information can distinguish the (b) diagram (2 Level 2 nodes, 1 Level 2 node) from the (a) diagram. (i) One type of additional information is from multiple deletions. The alternative input AI-X_1_ in the double deletion background x_2_Δ x_3_Δ is 0 for diagram (a), but 1 for diagram (b). (ii) A second type of information is from quantitation of the output assuming equal contribution from each path. (iii) A third type of information is measuring the activity at the individual nodes. Here we use activation information from node 2 (@X2) and node 3 (@X3) to distinguish the two 3-OR diagrams.

Because there may be many possible *Min+x* directed graphs (arrow diagrams) that are consistent with a given k-OR relationship graph, additional information is needed to distinguish among these possible graphs so that one or a few arrow diagrams are identified. Here we propose three types of strategies to collect more information (Figure 4):

1. *d-Deletions*. Instead of deleting a single gene, one can simultaneous delete *d* genes (d-deletions). It is possible to resolve a k-OR graph by making all possible 2, 3, 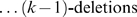 for each AI. This approach is only feasible for small *k* (e.g. 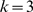), but we do expect *k* to be small for many biological signaling networks.
2. *Quantification of output*. Instead of converting the output into a Boolean value, one can take greater advantage of the continuous output value by treating the graph as a flow network. Then it is possible to evaluate different arrow diagram topologies according to the quantitative fit of the output data generated from the flow network of a given diagram with the actual data in the AIs-Deletions matrix.
3. *Individual node read-outs*. Instead of a single output node that is the sole read-out for the system, one can develop read-outs for each node, e.g. measuring the phosphorylation state of a protein. Then an AIs-Deletions submatrix can be constructed for each node resulting in a dramatic increase in information. The key is that it does not have to be done for all nodes, but only for one representative node in the C-node subgraph. Thus, in the k-OR case, there would be a 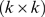 submatrix for each of the *k* C-nodes.

As an example, we consider a 3-OR relationship graph. There are two possible minimal arrow diagram representations for this case (Figure 4A). Using each of the three strategies it is possible to distinguish between these two classes (Figure 4B). We also point out that SIGNAL-AID was able to reconstruct a 3-OR case without additional information using information of the first row (natural input) of the AIs-Deletions matrix.

### Test Cases

We created test cases in which we took an arrow diagram from the literature and deconstructed a hypothetical Boolean AIs-Deletions matrix (e.g. Figures S1–S5). We then applied the algorithm to reconstruct the original diagram from the matrix. In the cases in which the maximum numbers of OR edges were 2 (2-OR) or 3 (3-OR), the program was able to reconstruct the diagram without additional information. In k-OR examples in which *k*>3, there were multiple possible diagrams that could be distinguished only by additional information (Text S1).

### Pilot Study: Yeast Mating Signaling System

The mating signaling network in budding yeast is one of the best characterized signal transduction systems [25]. Haploid **a**-cells respond to the extracellular input α-factor to mate with α-cells. Transcriptional activation of mating-related genes, formation of mating projections, and fusion of the two opposite mating type cells are involved in this process. The pathways in the mating signaling network have been determined by genetic, biochemical and molecular biological approaches in the late 1980s and early 1990s ([26]–[31], reviewed in [3]). Activation of gene expression occurs through the following pathway: α-factor → Ste2p → [Gpa1p/Ste4p/Ste18p] → Ste5p → Ste11p → Ste7p → Fus3p OR Kss1p → Ste12p → Transcription of mating-related genes.

**Figure 5:**
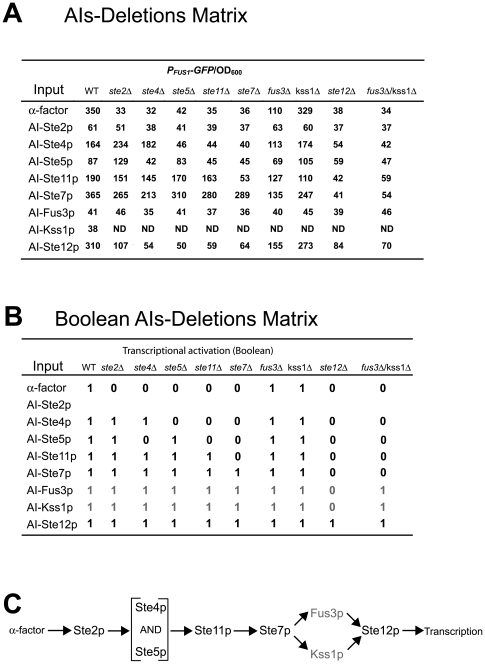
An AIs-Deletions matrix in the mating signaling transduction pathway in budding yeast. (A) AIs-Deletions matrix of transcriptional activation in the α-factor transcription pathway. We measured (t = 24 h) the fluorescence transcriptional read-out from cells containing each combination of an input (i.e. AIs) and a strain background (i.e. deletions). *P_FUS1_-GFP*/OD_600_ values were averaged from at least three measurements. ND indicates not determined. (B) Boolean AIs-Deletions matrix. The original transcription matrix was converted into a Boolean matrix by applying a threshold to each normalized GFP value. Then a 3-node consistency check was applied to fill-in missing data and to correct inaccurate data. For the AI-Fus3p and AI-Kss1p rows (gray), we show these filled-in consistency values because AI-Fus3p and AI-Kss1p were non-functional. (C) The arrow diagram for the yeast mating pathway reconstructed using the SIGNAL-AID program and the Boolean matrix in Figure 3B. This reconstruction required data from the *fus3Δ kss1Δ* double mutant strain shown in (A).

In this study, we focused on 8 signaling proteins of the α-factor transcription pathway: Ste2p, Ste4p, Ste5p, Ste11p, Ste7p, Fus3p, Kss1p and Ste12p. We prepared alternative inputs for the eight signaling molecules and monitored activation of the integrated transcriptional reporter *P_FUS1_-GFP* (Figure 5, details in Materials and Methods). We used the inducible GAL1 promoter to overexpress wild-type or constituitively-active versions of the genes. This approach successfully reconstructed the yeast mating arrow diagram including the parallel signaling by Fus3p and Kss1p.

We explored a flexible threshold scheme to convert the yeast mating transcription AIs-Deletions matrix into a Boolean matrix instead of using a fixed threshold value that produced inconsistencies in the resulting Boolean AIs-Deletions matrix. The main issue was that some AIs were stronger than others and so the threshold had to be calibrated appropriately. We devised the following threshold procedure that did not produce any inconsistencies.

If the value of *P_FUS1_-GFP*/OD_600_ was below 50, then the Boolean element was 0 (non-response); if the value of *P_FUS1_-GFP*/OD_600_ was above 60, then the element was 1 (response). Because of the weak activation properties of some AIs, we had to institute additional rules for values between 50 and 60. If it was 80% of the wild-type value, then the Boolean element *b_ij_* = 1, else *b_ij_* = 0. The AI value in the wild-type background was considered the reference value. For AI-Ste12p, we used the value of AI-Ste12p in the *mfa2Δ* strain as the reference value (60). We used this scheme to order the *fus3Δ kss1Δ* double deletion in the pathway.

**Figure 6:**
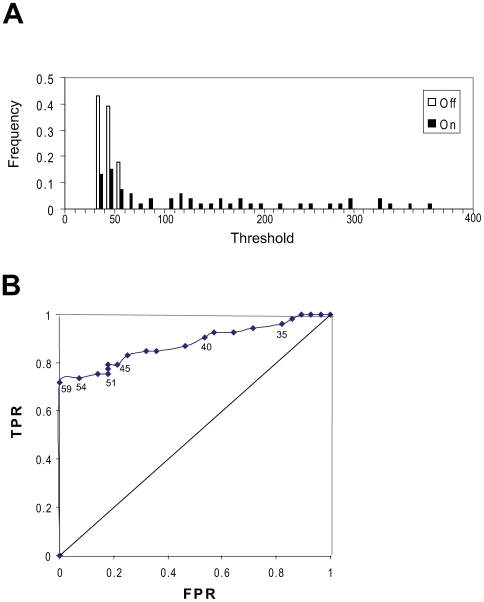
ROC (Receiver Operating Characteristic) analysis of threshold assignment to the AIs-Deletions matrix of the mating signaling pathway. (A) Histogram of the AIs-Deletions matrix values for the mating pathway example. Values were placed in bins of size 10. The proportion of the total number of on and off values are indicated for each bin. (B) The ROC curve for the experimental data of the AIs-Deletions matrix of the mating signaling pathway. This curve represents the false positive rates (FPR) and true positive rates (TPR) for a range of threshold values. FPR is defined as FP (False positive)/(FP + TN (true negative)), and TPR is defined as TP (true positive)/(TP + FN (false negative)). Selected threshold values are shown on the curve.

The value of the threshold can have a very important effect on the results. A histogram of the output values in the mating pathway AIs-Deletions matrix revealed a large cluster of values centered between 30 and 40 that represents mainly “off” responses with a few “on” responses (Figure 6A). To assess the fraction of incorrect classifications produced by different thresholds, we plotted the ROC (Receiving Operating Characteristic) curve for this AIs-Deletions matrix (Figure 6B). The TPR (true positive rate) is equivalent to sensitivity and the FPR (false positive rate) indicates specificity. Examining the histogram identified the range of values from 50 to 60 as a good place to put the threshold because that is the location of the tail of the cluster, and the ROC curve showed that threshold values in this range produced both specificity and sensitivity. Thus, it is possible to pick good threshold values *a priori*. Finally, as we describe in the robustness section, we have developed an error correction strategy that results in a perfect classification of response and non-response for this example.

### Two-Node (n = 2) and Three-Node (n = 3) Relationships

Here we describe our detailed analysis of two-node and three-node relationships. The three-node analysis was used as the basis of our three-node consistency check described in the next section on the robustness of the method to inaccurate and missing data.

For a signaling network containing *n* species, there are many possible arrow diagrams, pairwise relationship graphs, and AIs-Deletions matrices. It is instructive to examine all possible cases for small *n*. Here, we define *N_max_* as the maximum number of Boolean AIs-Deletions matrices, and *N* as the number of logically possible Boolean AIs-Deletions matrices (defined below). 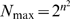, where *n^2^* represents the number of elements in the 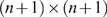 Boolean AIs-Deletions matrix minus the elements in the first column and the diagonal, which are all 1's by the definition of an AI.

**Figure 7:**
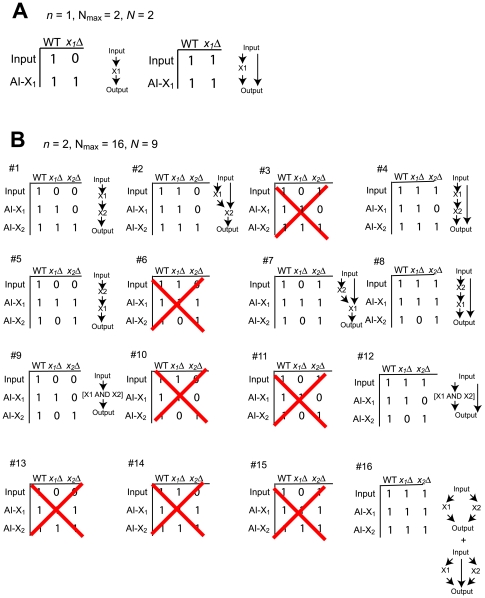
One-node and two-node Boolean AIs-Deletions matrices and signaling arrow diagram structures. (A) One species signaling system. There are two possible Boolean AIs-Deletions matrices in a one-node system. Both the matrices and their associated arrow diagrams are shown. (B) Two species signaling system. There are 16 Boolean AIs-Deletions matrices, however, 9 of them are logically possible. Those Boolean AIs-Deletions matrices and their corresponding arrow diagrams are shown.

A self-consistent or logically possible Boolean AIs Deletions matrix is one that can be converted into a signaling arrow diagram. When *n = 1*, *N_max_ = N = 2* (Figure 7A). When n = 2 (two-node diagrams), *N_max_ = 16*, however, the number of self-consistent AIs-Deletions matrix *N = 9* (Figure 7B) because there are several AIs-Deletions matrices that are not logically possible. For example, matrix number three is not self-consistent because X_1_ is downstream of the input, and X_2_ is downstream of X_1_, and yet X_2_ is not downstream of the input. These pairwise relationships result in a contradiction and cannot be represented as an arrow diagram.

**Figure 8:**
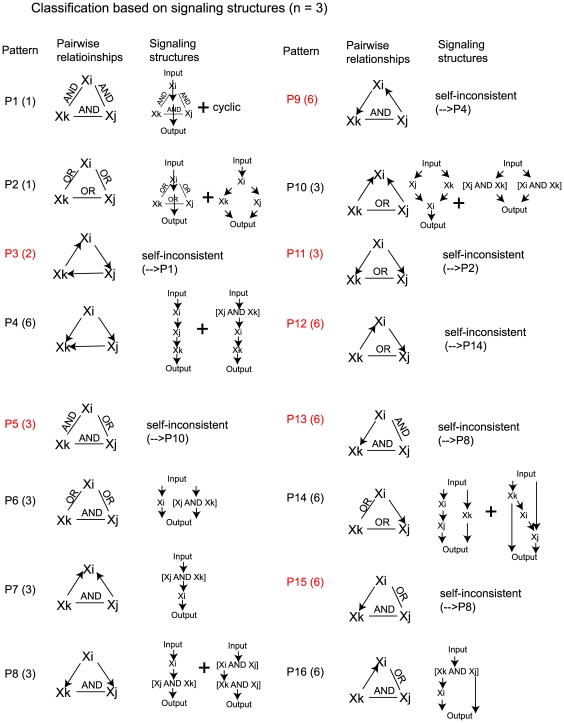
Three-node Boolean AIs-Deletions matrices and signaling arrow diagram structures. The three species signaling system gives rise to 64 pairwise relationship graph structures. These 64 structures could be grouped into 16 relationship patterns labeled P1 to P16; the number of permutations (i.e. permuting node labels (i, j, k)) for each pattern is shown in parentheses. The arrow diagram signaling structures for each pattern are shown next to the pattern. The red patterns are not self-consistent and cannot give rise to an arrow diagram.

When *n* = 3 (three-node diagrams), *N_max_* = 512. Here, there is greater complexity, and we focus on the relationships among the three nodes (64 distinct), and not on the relationships between the nodes and the input (8 possibilities). We group the 64 AIs-Deletions matrices into 16 patterns based on the structure of the pairwise relationship graphs (Figure 8). The three molecules are represented as (X_i_, X_j_, X_k_), and the indices (i, j, k) are assigned the values (1, 2, 3), and can be permuted for each pattern. Thus, we can enumerate how many permutations are in each signaling structure pattern. 9 of 16 signaling structure patterns were self-consistent, and 6 of the 9 consistent patterns gave rise to more than one signaling arrow diagram (i.e. P1, P2, P4, P8, P10, and P14).

The three-node example provides insight into the richness of the arrow diagram network structures that can arise from the AIs-Deletions analysis. Classic epistasis analysis focused on ordering linear pathways; the AIs-Deletions analysis is able to reconstruct networks containing nodes with complex branching patterns.

### Robustness of Method to Missing and Inaccurate Data

In any functional genomics strategy, one expects a significant error rate because of the high-throughput data collection. Thus, it was important to explore the tolerance of the alternative inputs approach to missing and inaccurate data. The key insight is that one can take advantage of 3-node pairwise relationships to fill-in missing data or correct inaccurate data; not all 3-node relationships are self-consistent in terms of interpretation into an arrow diagram. For example, given X_i_ → X_j_ and X_j_ → X_k_, then the three pairwise relationships X_k_ → X_i_ (cycle), X_k_ AND X_i_, and X_k_ OR X_i_ are not possible; X_i_ → X_k_ is the sole consistent relationship. Indeed, only 32/64 3-node patterns are self-consistent (Figure 8).

**Figure 9:**
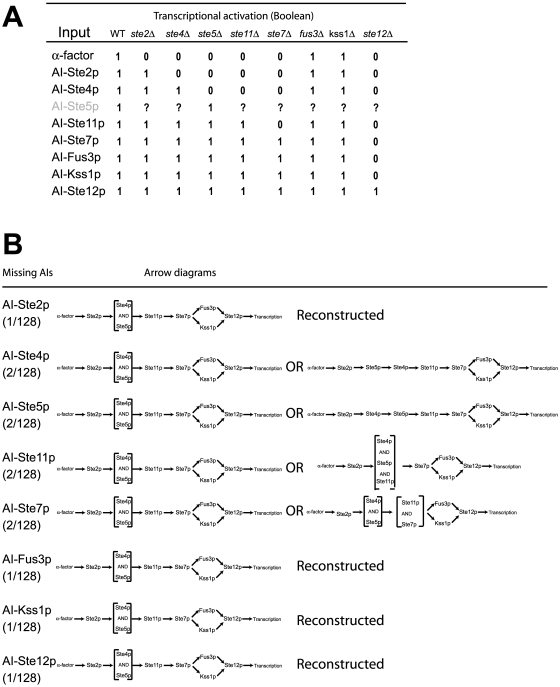
Robustness analysis for missing AIs. (A) Yeast mating AIs-Deletions matrix missing the AI-Ste5p row (fourth row). This matrix represents the situation in which data is missing because of a non-functional alternative input. 1's are placed in the columns for wild-type (WT) and *ste5Δ* because of the definition of an alternative input, and question marks are placed at the other positions in the AI-Ste5p row. (B) Reconstructing the yeast mating arrow diagram using a Boolean AIs-Deletions matrix missing an alternative input. We removed each of the alternative inputs and then attempted to reconstruct the arrow diagram using the three-node relationships (the four-node relationships were also used for missing AI-Fus3p and AI-Kss1p) to fill-in the missing matrix elements. There were either one or two possible arrow diagrams that are listed next to each missing AI.

Missing data is most likely to arise from non-functional AIs. In the yeast mating example, we examined what would happen if one AI were non-functional. In Figure 9A, we see that the AI-Ste5p row is undetermined. Using the 3-node relationships we can fill all of the entries in the row except for the AI-Ste5p ste4Δ element, which could be 0 (Ste4 AND Ste5) or 1 (Ste4 → Ste5). Thus, we were able to reconstruct the arrow diagram to one of two possibilities (originally there were 2^7^ or 128 possibilities). In Figure 9B, we show the possible reconstructed arrow diagrams if each AI were missing.

For two missing AIs in the yeast mating example, one can apply the same reasoning as above (data not shown). However, if both AI-Fus3p and AI-Kss1p were non-functional (as was the case), then they cannot be positioned in the pathway without information from the double deletion *fus3Δ kss1Δ* strain used in combination with the AIs. However, using the *fus3Δ kss1Δ* data, we were able to reconstruct the mating signaling network even without information from AI-Fus3p and AI-Kss1p (Figure 5).

We encountered the issue of inaccurate data, when we attempted to select a threshold for converting the real-valued AIs-Deletions Matrix (Figure 5A) into the Boolean AIs-Deletions matrix (Figure 5B). No single threshold value produced the correct Boolean matrix for the network as described above; the best values between 50 and 60 resulted in 3 to 4 incorrect matrix entries. However, these incorrect entries could be identified because they gave rise to inconsistent 3-node relationships. Making changes to resolve these inconsistencies resulted in the correct Boolean AIs-Deletions matrix and arrow diagram (Figure 5C). Finally, we found that using a flexible relative threshold that was adjusted to the strength of the AI reduced the number of inconsistencies and so was superior to a fixed threshold (see above).

### Applying the Alternative Inputs Approach to Functional Genomics

The Alternative Inputs approach might be applied to other signaling system to complement existing functional genomics methods through the process described in Figure 1. The first step would be to pre-screen for candidates that are likely to be involved in the particular input-output system. Using the natural input and the deletion strain library, one could identify gene deletions that reduce or increase the output significantly. One could then investigate all possible AIs-Deletions combinations of these candidates. Then, one could get the arrow diagram for the signaling network using SIGNAL-AID.

As we encountered in the pilot study, the greatest technical hurdle is making functional alternative inputs for all of the genes. In some cases, one can overexpress the wild-type form of the gene, for other signaling molecules (e.g. G-proteins), a well-conserved mutation can produce the constitutively-active form, and in other cases, one can take advantage of information in the literature to design the proper AI (e.g. AI-Ste7p). In addition, as described above, this methodology can tolerate missing AIs to a certain extent.

## Discussion

### Reverse Engineering a Signaling Network Using Alternative Inputs

A central idea of this paper is the concept of the AIs-Deletions matrix that summarizes outputs of all combinations of gain-of-function mutations (AIs) and loss-of-function mutations (deletions). We transformed real data into Boolean data, extracted information from these genetic interactions about functional ordering and logical relationships (AND and OR), provided an algorithm to construct a standard arrow diagram from an AIs-Deletions matrix, and implemented the algorithm in software named SIGNAL-AID.

Many reverse engineering techniques have been developed to reconstruct biological networks [19], [20], and our approach can complement these approaches to provide arrows and logics (AND or OR) to biological network diagrams in a more direct fashion based on classic epistasis data. We used standard brute-force matrix sorting algorithms to deduce the arrow diagram from the AIs-Deletions matrix (Materials and Methods), and this technique did not require statistical inference. Whereas the elegant synthetic lethality and E-MAP approaches relied on loss-of-function/loss-of-function mutant combinations, the AIs approach uses the gain-of-function/loss-of-function combinations of classic epistasis analysis.

This paper is most similar to the results of Zupan et al. [32]. They developed the GenePath program to construct genetic networks from mutational data. They defined three “inference patterns”: (1) Influence, which loosely corresponds to our concept of an alternative input; (2) Parallelism, which captures aspects of the OR relationship; and (3) Epistasis, which is equivalent to the notion of upstream and downstream. However, we believe that our work represents the next stage of development for this research direction. First, we propose to perform systematic epistasis analysis in which every gene is used both as an alternative input and a deletion leading to the AIs-Deletions matrix. Second, our theoretical framework defines all possible pairwise relationships, including AND relationships, which is missing from their treatment. Third, our definition of an OR relationship is richer than their concept of parallelism. For example, in the mating example, Fus3 and Kss1 would not be considered in parallel pathways according to their definition because the phenotype of the *fus3Δ kss1Δ* double mutant is the same as the single mutant deletions. Fourth, in the k-OR groups there are more complex network architectures than parallel pathways e.g. branched pathways of a 4-OR architecture. Fifth, we developed a method for checking for inconsistencies and filling-in missing data using a 3-node consistency check. Thus, we believe that this work is an important extension and systematization of the pioneering results of Zupan et al. [32].

Up to now, classic epistasis analysis has been done by hand. What are the benefits of automating this task? (1) To handle large (i.e. genome-scale) problems. Even for a linear pathway, manually ordering 100 genes would be arduous by hand. (2) It would be difficult to deconvolve branched pathways (i.e. k-OR relationships) by hand. If *k* is small, then the computer can handle this situation automatically. If *k* is large, then the computer can at least break the graph up into more manageable subgraphs and aid in enumerating feasible arrow diagrams consistent with the k-OR relationships. (3) The program can identify inconsistencies in the Boolean AIs-Deletions matrix and possibly resolve these inconsistencies. (4) In the case of missing or inaccurate data, the computer can generate a list of possible arrow diagrams that best correspond to the data.

### Future Directions

Regulation often modulates an output quantitatively and dynamically instead of turning it off or on. In our treatment, the genes involved in a positive feedback loop form a mutual AND relationship, but we cannot distinguish between a positive feedback loop and a complex, which will also have a mutual AND relationship among components. Isalan has pointed out that using at least two time points instead of one time point can resolve the paradox of representing negative feedback in gene networks [33]. One future direction would be to develop more output categories (e.g. high/medium/low/off) as well as incorporating information about timing (early/late). In addition, the genetic perturbations could encompass different degrees of expression. In this manner, we can begin to bridge the gap from arrow diagrams to more quantitative models of the system, and thus start to handle feedback loops.

The current method uses a 3-node consistency check to fill-in missing data and correct inaccurate data. However, this procedure will not work if there is too much experimental uncertainty. In the future, we would like to develop algorithms to enumerate and rank arrow diagrams during this consistency check according to self-consistency, how well each diagram can explain the AIs-Deletions matrix data, and parsimony (i.e. minimum number of edges), thus leading to a confidence score.

In our current framework (SIGNAL-AID-v1), we demonstrated the potential complexity of OR-included systems. In k-OR situations with *k*>3, we showed that we need additional information such as d-Deletions, quantification of output, and individual node read-outs to specify an arrow diagram from the feasible k-OR diagrams. Among these methods, the quantification of the output does not require additional experiments, and can be developed into a model selection criteria. Briefly, in the simplest case, equal weight can be given to each arrow, and a flow diagram can be constructed to calculate the output value when different edges are removed by deletions. Then, each architecture can be ranked according to the quantitative fit with the real data (Figure 4B). A further description is beyond the scope of this paper, but in the future we plan to examine and test this approach on both simulated and real data sets.

### Conclusions

Here we have developed the theory, algorithms, and outlined the experimental methodology for performing systematic epistasis analysis to reverse engineer the arrow diagram for a signal transduction network that extends the epistasis analysis to more complex networks. We term our approach “Alternative Inputs” and we exploit the ordering and logical information from gain-of-function (AIs) and loss-of-function (deletions) mutant combinations. Our pilot study on the yeast mating signaling system highlights the robustness of the alternative inputs strategy, and motivates its application on a larger genome-wide scale by addressing important technical issues. In particular, the method can tolerate missing and inaccurate data. We believe that alternative inputs approach complements existing functional genomics methods with its more direct interpretation into an arrow diagram and has the potential to reveal numerous novel interconnections in signaling networks when applied to a wide range of signaling inputs and outputs in a variety of organisms.

## Materials and Methods

### Strains and Plasmids

Standard genetic techniques were performed according to [34]. Yeast strains and plasmids used in this study are listed in Tables S1 and S2, respectively.

The *P_FUS1_-GFP* reporter (*HIS5*-marked PCR fragment) [35] was targeted to the *HIS3* locus of the strain RJD863 by PCR-based gene integration to create the strain HTY028. Then, the *mfα1Δ* strain HTY064 was constructed by PCR-based gene disruption of HTY028. In this study, HTY064 was used as the “wild-type” strain, and all deletion strains were derived from HTY064 by PCR-based gene disruption.

We constructed the alternative inputs expression plasmids as follows. Genes in the α-factor transcription pathway (*STE2*, *STE4*, *STE5*, *STE11*, *STE11ΔN* (residues 344–717) *STE7*, *FUS3*, *KSS1*, and *STE12*) were amplified by PCR (Phusion polymerase, New England Biolabs), and then were inserted into the pYES2 vector (Invitrogen) to create the *GAL1* promoter-regulated constructs in a high-copy number plasmid. The *P_GAL1_-STE2^P258L S259L^* and *P_GAL1_-FUS3^I161L^* constructs were created using QuickChange II Site-Directed Mutagenesis Kit (Stratagene). See Table S2 for plasmid constructs.

### Mating Transcriptional Activity Assay

1.5 ml of the total 2 ml cell culture was harvested and resuspended in PBS. Then, 100 µl of cells was placed into a 96-well plate and transcriptional activation was measured without fixation. The OD_600_ of the cells in the PBS solution was also measured using a spectrophotometer. Mating transcriptional activity from a integrated genomic reporter gene (*P_FUS1_-GFP*) was assayed using a Gemini XS SpectraMAX fluorometer with the excitation at 470 nm and emission at 510 nm as described previously [35]. The GFP fluorescence (arbitrary units) was normalized to the OD_600_, and the *P_FUS1_-GFP*/OD_600_ values were averaged over at least three independent experiments.

### Description of SIGNAL-AID Program

Here, we provide an overview of the SIGNAL-AID program and the ConvertToArrowDiagram algorithm that converts the pairwise relationship graph into a signaling arrow diagram. This algorithm was implemented in the SIGNAL-AID program.

SIGNAL-AID

1. Input Boolean AIs-Deletions matrix *M*
2. Convert *M* into Pairwise Relationship Graph *G*
3. Perform 3-Node Consistency Check on *G*
4. Identify joint AND nodes
5. Construct Arrow Diagram: *D*  =  ConvertToArrowDiagram(*G_s_*, *OutputNode*)
6. Link together k-OR subgraphs with arrows using k-OR enumeration procedure (not included in version 1.0)

ConvertToArrowDiagram(*g_s_*, *PreviousDownstreamNode*)

1. If a subgraph *g_s_* is a single node, then connect *g_s_* to *PreviousDownstreamNode* with an “upstream of” arrow and Return.
2. Else if not single node, check if there is a most downstream node of *g_s_* i.e. node that is downstream of all other nodes in *g_s_*.
3.
  -  a. If Yes, label this node *DownstreamNode*, and connect with *PreviousDownstreamNode* with “upstream of” arrow, then ConvertToArrowDiagram(*g_s_* – {*DownstreamNode*}, *DownstreamNode*).
  -  b. If No, identify largest subgraphs *S_i_* sharing a common downstream node *C_i_*. If there are *k* such subgraphs record that the *k* subgraphs [*S_1_* … *S_k_*] share a k-OR relationship and connect to the *PreviousDownstreamNode*. Then, for each *S_i_*, ConvertToArrowDiagram(*S_i_*, *C_i_*).

The three-node consistency check procedure has a running time of *O(n^3^)*, *n*  =  number of nodes, and the ConvertToArrowDiagram procedure has a running time of *O(n^2^)*, which involves searching the AIs-Deletions Boolean matrix for 0's. This brute-force approach is necessitated by the need to identify k-OR subgraphs as described above. In OR-excluded diagrams, one could employ a standard matrix sorting algorithm like topological sort, which is *O(n + E)*, *E*  =  number of edges. At most, *n* is the number of genes in the genome (e.g. ∼6000 in budding yeast), but for most problems, we expect fewer nodes because one can identify relevant genes for a given input/output by appropriate prescreening experiments.

SIGNAL-AID is written in the scripting language of MATLAB and can be run on any platform within the MATLAB environment. The licensing is GPLv3, and the program completed the 24-node Insulin example in a matter of seconds.

## Supporting Information

Text S1 Supplementary Information “Reverse Engineering a Signaling Network Using Alternative Inputs”
(0.04 MB DOC)

Table S1 Yeast strains used in this study
(0.02 MB DOC)

Table S2 Plasmids used in this study
(0.02 MB DOC)

Figure S1 Reconstructing the arrow diagram for the p53 signaling pathway. (A) Boolean AIs-Deletions matrix for p53 pathway example. (B) Arrow diagram of system reconstructed with SIGNAL-AID program using the information from the AIs-Deletions matrix.
(0.85 MB EPS)

Figure S2 Reconstructing the arrow diagram for the G-protein pathway involved in Alzheimer's disease. (A) Boolean AIs-Deletions matrix for G-protein pathway involved in Alzheimer's disease example. (B) Pairwise relationship graph showing 3-OR relationship. (C) Arrow diagram of system reconstructed with SIGNAL-AID program using the information from the AIs-Deletions matrix.
(1.04 MB EPS)

Figure S3 Reconstructing the arrow diagram for the insulin signaling pathway. (A) Boolean AIs-Deletions matrix for insulin signaling pathway example. (B) Arrow diagram of system reconstructed with SIGNAL-AID program using the information from the AIs-Deletions matrix and simulated experimental data from an individual node read-out experiment involving the 4 C-node subgraphs in the 4-OR relationship (Figure S5).
(1.37 MB EPS)

Figure S4 Reconstructing the arrow diagram for the insulin signaling pathway - the output from SIGNAL-AID. (A) SIGNAL-AID returns a C-Node list consisting of Input Lists, OR Lists, and Output Lists, and a list of node pairs sharing an AND relationship. The AIs-Deletions matrix shown in Figure S3A was used as the input. (B) The signaling network produced by the information in (A). This signaling network contains a 4-OR cluster, and we investigate the different possible connectivity patterns of the C-nodes in Figure S5.
(1.23 MB EPS)

Figure S5 Reconstructing the arrow diagram for the insulin signaling pathway - AIs-node-readouts matrix. Some topological candidates for the connectivity of the C-nodes shown in Figure S4B are listed on the left. The topological graphs were reproduced from Figure 3. The corresponding AIs-node-readouts matrices are shown on the right. Here, the convention is that the rows contain the AIs from C-nodes shown in Figure S4B, and the columns contain the node-readout at each C-node. The resulting output values in these matrices can identify the correct connectivity of the C-nodes (Figure S3B).
(1.02 MB EPS)

Figure S6 Reconstructing the arrow diagram for the mating signaling pathway containing the repressor Gpa1p. (A) Simulated data for AI-Gpa1p (*gpa1Δ*) and Delta-Gpa1p (overexpression of Gpa1p) were added to the Boolean AIs-Deletions matrix shown in Figure 5B. (B) The arrow diagram for the yeast mating pathway reconstructed using the SIGNAL-AID program and the Boolean AIs-Deletions matrix in (A).
(0.93 MB EPS)
