# Correction: Reverse Engineering a Signaling Network Using Alternative Inputs

**DOI:** 10.1371/annotation/c9e68857-cd7e-4b1c-9765-04f66d8237d0

**Published:** 2009-12-11

**Authors:** Hiromasa Tanaka, Tau-Mu Yi

Figure 5 is incomplete. Please view the correct figure here:

**Figure 5:**
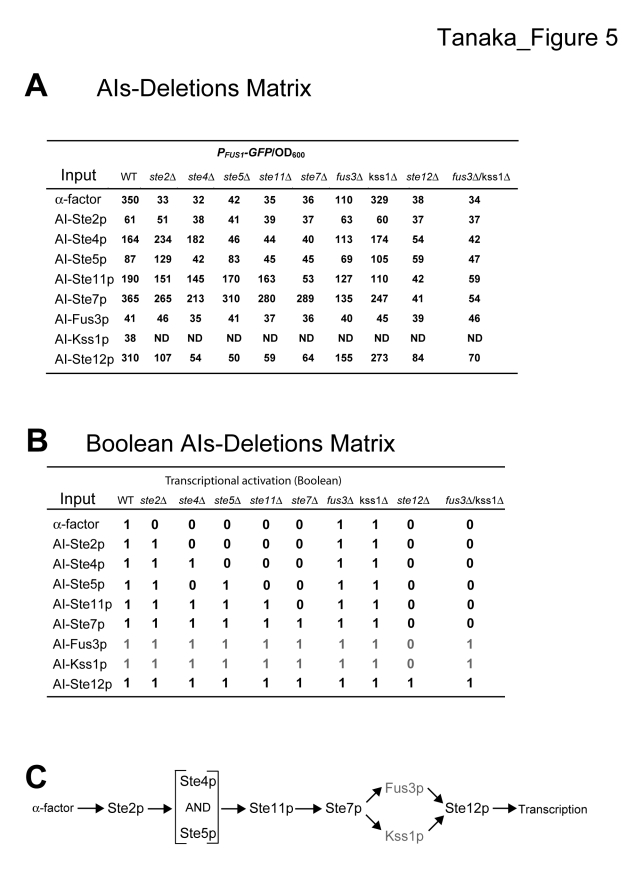
An AIs-Deletions matrix in the mating signaling transduction pathway in budding yeast.

