# Supplementary material for: Reverse Engineering a Signaling Network Using Alternative Inputs
Source: PLoS One. 2009 Oct 29;4(10):e7622. doi: 10.1371/journal.pone.0007622 (PMC2764141; doi:10.1371/journal.pone.0007622)
Supplement: Text S1 — Supplementary Information “Reverse Engineering a Signaling Network Using Alternative Inputs” (0.04 MB DOC) [file pone.0007622.s001.doc]

**Supplementary Information**

# Reverse Engineering a Signaling Network Using Alternative Inputs

Hiromasa Tanaka^1,2^ and Tau-Mu Yi^1,2^

^1^Department of Developmental and Cell Biology

^2^Center for Complex Biological Systems

University of California, Irvine, CA 92697 USA

### Description of signaling arrow diagram

Here we define a biological signaling arrow diagram to be a directed acyclic Boolean graph. A directed edge from X_1_ to X_2_ (X_1_ 🡪 X_2_) indicates that X_1_ activates X_2_ and that if X_1_ = 1, then X_2_ = 1. AI-X_1_ sets X_1_ = 1, and *x_1_Δ* sets X_1_ = 0.

A given activator can activate more than one target, resulting in a branch node (X_1_ 🡪 X_2_, X_1_ 🡪 X_3_). Such a branch node gives rise to parallel pathways and the OR pairwise relationship among nodes.

A given target can be activated by more than one activator, e.g. X_1_ 🡪 X_3_, X_2_ 🡪 X_3_. In this example, $X_{3}=X_{1}\vee X_{2}$. More generally, inference in the network proceeds by the rule that the target node equals the Boolean sum of the activator nodes.

We interpret a cycle in the arrow diagram as a positive feedback loop resulting in a mutual AND relationship among the nodes. We use an example to illustrate this interpretation. Let X_1_ 🡪 X_2_ 🡪 X_3_ 🡪 X_1_, and X_3_ 🡪 Output. Because of the positive feedback loop, then there is no signaling to the output if the loop is broken. As a result, every node is downstream of all other nodes i.e. AI-X_i_ x_j_Δ = 0 for all *i*, *j*. Thus, in the pairwise relationship graph, X_1_, X_2_, and X_3_ would be connected by AND edges (P1 in Figure 8). In our algorithm, the first step is to group mutual AND nodes together to create a joint AND node. In this manner, we can exclude cycles from consideration in the arrow diagrams allowing us to treat the diagrams as directed acyclic graphs that may contain a joint AND node potentially arising from a positive feedback loop.

# Reconstructing signaling network arrow diagrams using SIGNAL-AID

We tested the SIGNAL-AID program on various test cases (Figure S1-S5), and here we show 3 examples from the KEGG database. The first example (<http://www.genome.jp/kegg/pathway/hsa/hsa04115.html>) is the p53 pathway that contains 5 species and is a 2-OR case. The second example (<http://www.genome.jp/kegg/pathway/hsa/hsa05010.html>) is a G-protein pathway involved in Alzheimer’s diseases that contains 6 species and is a 3-OR case. The third example (<http://www.genome.jp/kegg/pathway/hsa/hsa04910.html>) is from the insulin signaling pathway and contains 24 nodes and is a 4-OR case (Figure S4). For this last example, we used simulated individual node read-out data to determine the minimal arrow diagram connectivity of the C-node subgraphs in the mutual 4-OR relationship (Figure S5).

We analyzed the robustness of this example to missing or inaccurate data. We systematically removed one AI and then determined the number of self-consistent topologies. We found that for the different missing AIs there were between 1 and 20 feasible architectures from a total number of 2^23^ possibilities by using the 3-node consistency check. In addition, in 10 trials, we randomly picked 10 elements in the AIs-Deletions matrix and explored all possible combinations of 0’s and 1’s. We found that there were between 1 and 20 feasible architectures from a total number or 2^10^ possibilities. Thus, for a large example the method shows robustness when the amount of missing/inaccurate data is small.

# Signaling repressors

In this study, we focused on signaling activators, however, signaling repressors can be incorporated into this framework by extending the concept of the AIs-Deletions matrix. In the simplest case, the deletion of the repressor would be the “alternative input” and the overexpression of the repressor would be the “deletion.” In the mating signaling system, Gpa1p is a good example of a repressor. The *gpa1Δ* strains are constitutively active [1,2], and so the deletion of Gpa1 acts as an alternative input. We considered the repressor Gpa1p using simulated data (Figure S6). We point out that Sst2p should not be considered as a repressor in the framework of the alternative inputs approach because *sst2Δ* strains are not constitutively active although the strain becomes supersensitive (enhanced signaling) to the natural input [3]; Sst2p would be considered a negative regulator. In addition, a protein, e.g. Gpa1p, may possess dual roles acting as a repressor for one output and as an activator for a different output [4]. We also considered repressors in the case of the insulin pathway (Figure S3).

## References

1. Dietzel C, Kurjan J (1987) The yeast SCG1 gene: a G alpha-like protein implicated in the a- and alpha-factor response pathway. Cell 50: 1001-1010.

2. Miyajima I, Nakafuku M, Nakayama N, Brenner C, Miyajima A, et al. (1987) GPA1, a haploid-specific essential gene, encodes a yeast homolog of mammalian G protein which may be involved in mating factor signal transduction. Cell 50: 1011-1019.

3. Chan RK, Otte CA (1982) Isolation and genetic analysis of Saccharomyces cerevisiae mutants supersensitive to G1 arrest by a factor and alpha factor pheromones. Mol Cell Biol 2: 11-20.

4. Guo M, Aston C, Burchett SA, Dyke C, Fields S, et al. (2003) The yeast G protein alpha subunit Gpa1 transmits a signal through an RNA binding effector protein Scp160. Mol Cell 12: 517-524.
