# Supplementary material for: Reverse Engineering a Signaling Network Using Alternative Inputs
Source: PLoS One. 2009 Oct 29;4(10):e7622. doi: 10.1371/journal.pone.0007622 (PMC2764141; doi:10.1371/journal.pone.0007622)
Supplement: Table S1 — Yeast strains used in this study (0.02 MB DOC) [file pone.0007622.s002.doc]

**Table S1. Yeast strains used in this study**

| **Strain** | **Genotype** | **Source** |
| --- | --- | --- |
| RJD360 | *MAT****a*** *can1-100 leu2-3-112 his3-11-15 trp1-1 ura3-1 ade2-1* | Ray Deshaies |
| RJD863 | RJD360 *bar1Δ::hisG* | Ray Deshaies |
| HTY064 | RJD863 *mfα1Δ::LEU2* *his3Δ::HIS3MX6-P_FUS1_-GFP* | This study |
| HTY136 | HTY064 *ste2Δ::KanMX4* | This study |
| HTY138 | HTY064 *ste4Δ::KanMX4* | This study |
| HTY145 | HTY064 *fus3Δ::KanMX4* | This study |
| HTY152 | HTY064 *fus3Δ::KanMX4 kss1Δ::HYGB* | This study |
| HTY158 | HTY064 *ste7Δ::KanMX4* | This study |
| HTY159 | HTY064 *ste11Δ::KanMX4* | This study |
| HTY160 | HTY064 *ste12Δ::HYGB* | This study |
| HTY167 | HTY064 *ste5Δ::HYGB* | This study |
| HTY172 | HTY064 *kss1Δ::HYGB* | This study |
