# Supplementary material for: Reverse Engineering a Signaling Network Using Alternative Inputs
Source: PLoS One. 2009 Oct 29;4(10):e7622. doi: 10.1371/journal.pone.0007622 (PMC2764141; doi:10.1371/journal.pone.0007622)
Supplement: Table S2 — Plasmids used in this study (0.02 MB DOC) [file pone.0007622.s003.doc]

# Table S2. Plasmids used in this study

| **Name** | **Description** | **Vector base** | **Source** |
| --- | --- | --- | --- |
| pHT002 | 2μ *URA3 P_GAL1_-STE2(P258L/S259L)* | pYES2 | This study |
| pHT003 | 2μ *URA3 P_GAL1_-STE4* | pYES2 | This study |
| pHT004 | 2μ *URA3 P_GAL1_-STE5* | pYES2 | This study |
| pHT006 | 2μ *URA3 P_GAL1_-STE11ΔN* | pYES2 | This study |
| pHT008 | 2μ *URA3 P_GAL1_-STE11ΔN-STE7* | pYES2 | This study |
| pHT010 | 2μ *URA3 P_GAL1_-FUS3(I161L)* | pYES2 | This study |
| pHT011 | 2μ *URA3 P_GAL1_-KSS1* | pYES2 | This study |
| pHT012 | 2μ *URA3 P_GAL1_-STE12* | pYES2 | This study |
